# Recurrent seasonal outbreak of an emerging serotype of Shiga toxin-producing *Escherichia coli* (STEC O55:H7 Stx2a) in the south west of England, July 2014 to September 2015

**DOI:** 10.2807/1560-7917.ES.2017.22.36.30610

**Published:** 2017-09-07

**Authors:** Noëleen McFarland, Nick Bundle, Claire Jenkins, Gauri Godbole, Amy Mikhail, Tim Dallman, Catherine O'Connor, Noel McCarthy, Emer O'Connell, Juli Treacy, Girija Dabke, James Mapstone, Yvette Landy, Janet Moore, Rachel Partridge, Frieda Jorgensen, Caroline Willis, Piers Mook, Chas Rawlings, Richard Acornley, Charlotte Featherstone, Sharleen Gayle, Joanne Edge, Eleanor McNamara, Jeremy Hawker, Sooria Balasegaram

**Affiliations:** 1 Health Protection Team (Fareham), Public Health England South East Centre, Fareham, United Kingdom; 2 These authors contributed equally to this article and share first authorship; 3 UK Field Epidemiology Training Programme, Public Health England, London, United Kingdom; 4 Field Epidemiology Services, National Infection Service, Public Health England, London, United Kingdom; 5 European Programme for Intervention Epidemiology Training (EPIET), European Centre for Disease Prevention and Control (ECDC), Stockholm, Sweden; 6 Gastrointestinal Bacteria Reference Unit, National Infection Service, Public Health England, London, United Kingdom; 7 Reference Microbiology Services, National infection Service, Public Health England, London, United Kingdom; 8 Gastrointestinal Infection Department, National Infection Service, Public Health England, London, United Kingdom; 9 Emerging Infections and Zoonoses, National infection Service, Public Health England, London, United Kingdom; 10 University of Warwick, Coventry, United Kingdom; 11 National Institute Health Research (NIHR) Health Protection Research Unit in Gastrointestinal Infections, London, United Kingdom; 12 UK Public Health Training Scheme, London, United Kingdom; 13 Public Health England South of England Region, Bristol, United Kingdom; 14 Bournemouth Borough Council, Bournemouth, United Kingdom; 15 Weymouth & Portland Borough Council and West Dorset District Council, Weymouth, United Kingdom; 16 Public Health Dorset, Dorchester, United Kingdom; 17 Food Water and Environmental Microbiology Laboratory, National Infection Service, Public Health England, Salisbury, United Kingdom; 18 Environment Agency, Wessex, Blandford, United Kingdom; 19 Animal and Plant Health Agency, Thirsk, United Kingdom; 20 Food Standards Agency, London, United Kingdom; 21 Public Health Laboratory, Health Service Executive, Dublin, Republic of Ireland

**Keywords:** New strain, STEC, outbreak, pets, cattle testing, Boot sock study, STEC 055

## Abstract

The first documented British outbreak of Shiga toxin-producing *Escherichia coli* (STEC) O55:H7 began in the county of Dorset, England, in July 2014. Since then, there have been a total of 31 cases of which 13 presented with haemolytic uraemic syndrome (HUS). The outbreak strain had Shiga toxin (Stx) subtype 2a associated with an elevated risk of HUS. This strain had not previously been isolated from humans or animals in England. The only epidemiological link was living in or having close links to two areas in Dorset. Extensive investigations included testing of animals and household pets. Control measures included extended screening, iterative interviewing and exclusion of cases and high risk contacts. Whole genome sequencing (WGS) confirmed that all the cases were infected with similar strains. A specific source could not be identified. The combination of epidemiological investigation and WGS indicated, however, that this outbreak was possibly caused by recurrent introductions from a local endemic zoonotic source, that a highly similar endemic reservoir appears to exist in the Republic of Ireland but has not been identified elsewhere, and that a subset of cases was associated with human-to-human transmission in a nursery.

## Introduction

Shiga toxin-producing *Escherichia coli* (STEC) is known to cause self-limiting diarrhoeal illness, sometimes bloody diarrhoea and complications such as haemorrhagic colitis and haemolytic uraemic syndrome (HUS) [1-3]. STEC O157:H7 is the most common serotype in England while the incidence of non-O157 STEC serotypes such as O26, O45, O103, O111, O121 and O145, some of which have been implicated in a number of outbreaks internationally [4-8], may be under-reported in the English national surveillance system run by Public Health England (PHE). This is due to the lack of general applicability of current culture-based detection methods [9,10].

### The signal

PHE South East Centre was notified in July 2014 of two children with HUS in the county of Dorset. Further cases, all living in Dorset, were identified during the following months, including an outbreak at a nursery. Faecal specimens tested at the local hospital laboratory were negative for STEC O157. Further testing at the Gastrointestinal Bacterial Reference Unit (GBRU) PHE, Colindale, London, identified the causative organism as STEC O55:H7 which carried the Shiga toxin gene (*stx*2) and the intimin-encoding gene *eae* (*E. coli* attaching and effacing). The combination of these virulence genes is associated with an elevated risk of HUS [11]. Phylogenetic analysis using whole genome sequencing (WGS) has indicated STEC O55:H7 to be the ancestor of the highly pathogenic clone STEC O157:H7 [12].

The reference laboratory in the Republic of Ireland (ROI) routinely send a sub-set of non-O157 STEC to GBRU for serotyping. A search of GBRU’s database, following detection of the STEC non-O157-related HUS cases in Dorset, revealed that nine cases of STEC O55:H7 had occurred in ROI between 2012 and 2014. However, prior to July 2014 STEC O55:H7 had not been isolated from human cases or animals in England. The outbreak starting in July 2014 was, therefore, the first known outbreak in England of STEC O55:H7 causing severe illness. Apart from human-to-human transmission, we considered three competing hypotheses based on established [13] STEC O55:H7 transmission pathways: (i) consumption of contaminated food or drinking water; (ii) specific recreational or environmental exposure; (iii) epizootic vector and/or general environmental contamination. The objectives of our investigation were case finding, investigating the source and controlling the outbreak.

## Methods

### Epidemiological investigations

#### Case definitions

A confirmed case was defined as an individual with STEC O55:H7 identified by culture from a faecal specimen or with antibodies to the lipopolysaccharide (LPS) of *E. coli* O55 identified by serology, and having spent time in Dorset in the 14 days before onset, between June 2014 and September 2015. A second inclusion criterion of being within 5 single nucleotide polymorphisms (SNPs) of the outbreak strain, where WGS was available, was added on 1 October 2015 to increase the specificity of the case definition by requiring genetic relatedness between isolates. Confirmed cases were further categorised as either symptomatic (with symptoms of gastrointestinal disease, haemorrhagic colitis, HUS or thrombotic thrombocytopenic purpura) or asymptomatic. Probable cases were individuals with the symptoms listed above and an epidemiological link to a confirmed case, or nursery cases who were culture negative on screening but *eae* positive on PCR.

Case finding is described under microbiological investigations, below.

#### Exposure histories

National enhanced surveillance for STEC at PHE includes collection and analysis of standardised microbiological, demographic, clinical, and exposure data on all cases [10]. We extended the questionnaire [14] to collect detailed histories from symptomatic cases about places visited, activities undertaken and food consumed in the 14 days before symptom onset. This was an extension of the usual 7-day period used for STEC O157 investigations since the incubation period for STEC O55 is unknown and we wanted to identify all potentially important exposures. Asymptomatic cases were also followed up using the same enhanced questionnaire with a focus on activities during the period beginning 14 days before the onset of symptoms of the associated primary, symptomatic case.

We used an iterative re-interviewing process, re-contacting previous cases for direct questioning on all new exposures that were reported by later cases. Re-interviews included extended food consumption histories, review of one-month shopping history and exposure to local events, venues, the outdoor environment, animals and pet food. Staff and parents in a nursery cluster completed a questionnaire on illness, nursery attendance, activities and staff roles.

We undertook network mapping to visualise any common exposures between cases using i2 Analyst’s Notebook (IBM Corporation, New York, United States (US)).

### Environmental investigations

#### Food, water, environmental and animal samples

Environmental Health Officers (EHOs) inspected and collected food, water and environmental samples from venues visited by cases which included a restaurant chain, mobile food vendor, public gardens and a lily pond. Additional environmental samples were collected using standard samples and swabs, faecal pots and ‘boot socks’, a previously described method of sample collection originally conceived to detect *Campylobacter* present in the environment [15]. These additional samples were collected from cases’ gardens, parks and other outdoor areas visited them, bird sanctuaries and local cattle grazing areas. Flood prone areas were also targeted, focusing on areas with wildlife activity and those downstream of cattle populations, as identified by agricultural census data. Waterways and surface water runoff were sampled by the national Environment Agency Wessex office, Blandford, Dorset.

After a positive cat faecal sample had been obtained, we retrospectively collected faecal specimens from pet dogs and cats of all cases from July 2014 onwards. Seventeen faecal specimens were taken from goats, cattle and horses at a petting farm, where a veterinary risk assessment was undertaken.

#### Food chain analysis

The Food Standards Agency (FSA) carried out supply chain investigations linked to food consumed by cases and pets. Not all food consumed was traced back; instead the focus was on identifying retail distribution local to the Dorset area that differed from supply to the rest of the country.

#### Hydrological analysis

We identified the dates of flooding from online media reports for the period December 2014 to September 2015 for a stream that flows through a popular public garden before discharging into the sea in Bournemouth. Additional theoretical flooding events in this period were identified from the 99th percentile of the distribution of measured stream depth data but for which there were no online media reports. For primary cases, resident in or having visited Bournemouth before illness, we calculated the delay between the dates of flooding events and symptom onset to identify plausible temporal associations between flooding and illness.

### Microbiological investigations

#### Case finding

Prior to the identification of STEC O55:H7 as the aetiological agent of this outbreak, serum samples from initial cases with HUS that were culture negative for STEC O157 were assessed for the presence of antibodies to the LPS of non-O157 serogroups (O26, O55, O103, O111, O128 and O145) at GBRU in PHE Colindale [16]. Faecal specimens were tested using PCR [17] at GBRU for the presence of *stx* genes and cultured on MacConkey, Sorbitol MacConkey (SMAC) and cefixime-tellurite SMAC (CTSMAC) agar. For all positive specimens, 10 colonies were retested using the same PCR. Those colonies testing positive for *stx* were identified biochemically, serotyped and characterised by additional PCR assays [17]. STEC guidelines [18] recommend testing faecal specimens for non-O157 STEC from HUS patients, cases of bloody diarrhoea where STEC is suspected, and *stx* PCR-positive faecal specimens at the local hospital laboratory. All close contacts of cases were screened via testing of serum and/or faecal specimens, including all children and staff in a nursery cluster.

From December 2014 to January 2015 and again from June 2015, active case finding included screening of faecal specimens from all individuals with bloody diarrhoea by Dorset laboratories, with prospective referral of negative specimens to GBRU for additional testing. In August 2015, enhanced testing was introduced in one Dorset laboratory and by October 2015, this was replicated in the remaining two laboratories and the PHE Specialist Laboratory in Southampton. This involved a 12-week extended testing regime, which analysed specimens from both primary and secondary care, with all diarrhoeal specimens tested using an additional MacConkey Agar with Sorbitol (SMAC) agar plate. Any non-sorbitol fermenting isolates of *E. coli* were sent to GBRU for further characterisation and typing.

#### Food, water and environmental specimens

Animal faecal specimens were analysed by GBRU using the same protocol as for human faecal specimens. All other samples were analysed by PHE’s Food, Water and Environmental (FWE) Microbiology laboratories. At FWE, real-time PCR was used to examine samples for the presence of STEC based on CEN/ISO TS 13136 [19]. Water samples (up to 1 L) were filtered and filtrates enriched in 100 mL Modified Tryptone Soya Broth (mTSB). Bootsocks were immersed in 250 mL mTSB and swabs were immersed in 90 mL mTSB. Enrichment broths that were PCR-positive for *stx* were sub-cultured onto MacConkey and SMAC agar and up to 50 colonies retested using the same PCR assay. Any STEC strains isolated were sent to GBRU for further characterisation.

#### Whole genome sequencing

All isolates of STEC O55:H7 from this outbreak cultured from 24 human and two animal faecal specimens together with 11 background STEC O55:H7 isolates from ROI contained within the PHE archive were whole genome sequenced by PHE Genome Sequencing Unit using Nextera library preparation on the Illumina HiSeq 2,500 run in fast mode according to the manufacturers’ instructions [20]. All sequences were mapped against the sequence of the *E. coli* O55:H7 reference strain CB9615 (GenBank accession number: CP001846.1) using BWA-MEM [19]. SNPs were identified using GATK2 [21] in unified genotyper mode. Core genome positions that had a high quality SNP (> 90% consensus, minimum depth 10x, MQ ≥ 30) in at least one strain were extracted and RaxML [21] used to derive the maximum likelihood phylogeny of the isolates under the GTRCAT model of evolution. FASTQ reads from all sequences in this study can be found at the PHE Pathogens BioProject at the National Center for Biotechnology Information (accession number: PRJNA315192).

### International investigations

The public health team in the ROI administered the PHE STEC questionnaire to earlier Irish cases and provided additional information on demographics and shared exposures of these cases. Postcodes of the primary cases in Dorset and the counties where infected individuals reside in ROI were taken as points of reference to examine both large scale migration and smaller regional movements of different migratory bird species using the British Trust for Ornithology mapping tool [22]. Live cattle movements between ROI and Dorset in 2013 and 2014 were mapped by date of movement and the postcode of the recipient farms/markets.

## Results

### Epidemiological investigation

**Figure 1:**
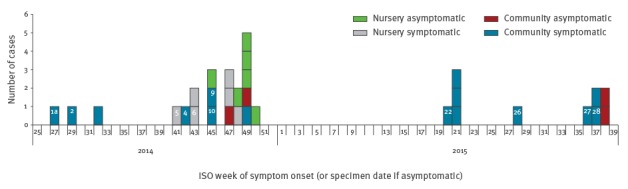
Confirmed cases of Shiga toxin-producing *Escherichia coli* 055:H7, Dorset, July 2014−September 2015 (n=31)

From July 2014 to September 2015 we identified 31 confirmed cases; 28 were associated with six epidemiological clusters (Table), with one cluster (family B) arising due to two (co)primary cases with symptom onset on the same day. Symptom onset was seasonal, occurring between July and November 2014 and May to September 2015 (Figure 1). There were three probable cases associated with the nursery and no probable community cases.

**Table:** Characteristics of confirmed cases of Shiga toxin-producing *Escherichia coli* O55:H7, Dorset, England, June 2014−September 2015 (n=31)

Variable	Description	All cases	Children (0–4 years)	Children (5–17 years)	Adults (18–69 years)
**Total number**		**31**	**16**	**4**	**11**
**Symptomatic^a^**	Yes	21	12	3	6
	No	10	4	1	5
**HUS**	Yes	13	9	2	2
	No	18	7	2	9
**Sex**	Male	13	8	2	3
	Female	18	8	2	8
**Age (years)**	Mean (SD)	16 (19)	2.2 (1.1)	9.8 (3.3)	39 (14)
	Median (range)	4 (0–69)	2 (0–4)	9.5 (6–14)	35 (25–69)
**Epidemiological cluster**	None^b^	3	1	1	1
	Family A	2	1	0	1
	Nursery	12	9	0	3
	Family B	5	2	1	2
	Family C	4	1	1	2
	Family D	2	1	0	1
	Family E	3	1	1	1

HUS: haemolytic uraemic syndrome; SD: standard deviation.

^a^ Symptoms included HUS (13/21), bloody diarrhoea (3/21) and diarrhoea (5/21).

^b^ Case numbers 1, 4 and 26 were not associated with epidemiological clusters.

ISO: International Organisation for Standardisation.

^a^ Case numbers of (co)primary cases shown.

Of 21 cases symptomatic with HUS, bloody diarrhoea or diarrhoea (21/31), 18 were confirmed on faecal culture and three by serology. Of the 10 asymptomatic cases (10/31), six were culture-positive and four were identified by serology. Twenty of the 31 cases were children, 16 were below the age of 5 years and 11 of the 20 children developed HUS (Table). Across all cases, HUS was more common (13/31) than expected for STEC O157 (172/3,323; 5%, chi-squared p < 0.0001) [2].

The network analysis revealed nine of the 10 (co)primary cases had domestic contact with cats (5/10) and/or dogs (5/10). In addition to domestic animal contact, the only common factor among the (co)primary cases was residing in or around, or having links to, Bournemouth and Weymouth in Dorset.

#### Nursery cluster

Twelve cases (of whom six were symptomatic) were associated with a nursery (Figure 1, Table), including staff, children and their household contacts. Twelve staff members in the nursery responded to the staff questionnaire (12/30), revealing a history of diarrhoea in three screen-negative staff in the previous month including one with a family connection to a confirmed case. Questionnaires on nursery children had a poor response rate of 14% but identified a child that may have been previously symptomatic. Six cases in children (two symptomatic) were identified from the 99 of 112 staff members and children screened. An additional three children were identified who were culture-negative but PCR positive for the *eae* (intimin) gene only, meaning they were defined as probable cases at the point of screening. One of these three children had been previously symptomatic and had a delay of ten days between symptom onset and specimen collection.

The delay between symptom onset and specimen collection for the confirmed nursery cases ranged from 0 to 9 days (mean: 5 days). The optimum time for testing is as soon as possible after onset however 5 days is still a satisfactory timeframe as PCR is able to detect dead bacteria. The investigation did not reveal any possible food or environmental sources of transmission at the nursery and no epidemiological links were identified between the primary nursery and primary community cases.

### Environmental investigation

#### Food, water, environmental and animal samples

No STEC O55:H7 was isolated from over 100 food, water and environmental samples or from 17 animal samples from the petting farm. STEC O55:H7 was isolated from a cat faecal specimen, taken from a concrete path outside the home of the primary case in Family C (Case 22). The pet cat of this household had been ill but tested negative. All pets of (co)primary cases tested retrospectively following this finding yielded negative results but the faeces of a symptomatic pet cat, tested prospectively, was positive for STEC O55:H7. This cat belonged to case 26, who was not associated with an epidemiological cluster, and the cat’s illness preceded symptom onset in the case. These two positive cat specimens were taken ca 4.5 km and two months apart.

#### Food chain analysis

The food chain analysis did not identify any product lines that were sourced specifically for retailers in the Dorset area. No links were identified between the premises investigated, including two nurseries, two petting farms, a local butcher, an abattoir, a café, takeaway restaurants, mobile food vans and a national chain of restaurants. There were no significant commonalities between food items consumed by the pets and all pet food brands reported had national distribution networks.

#### Hydrological data analysis

Four media reports of flooding events and two additional theoretical flooding events were identified during the period from December 2014 to September 2015. After excluding one theoretical event which occurred during January 2015 when there were no cases, the remaining five flooding events occurred 1–9 days (median: 8 days) before symptom onset among five of the six (co)primary cases linked to Bournemouth. Three of these cases reported having visited the public gardens and/or Bournemouth beach in their exposure histories.

### Microbiological investigation

#### Case finding

The outbreak strain cultures were serotyped as STEC O55:H7, tested positive for the presence of *eae* (intimin), had a *stx* subtype profile that was *stx2a* only and appeared as non-sorbitol fermenting colonies on SMAC but failed to grow on CTSMAC. Only one case (Case 26, who was not associated with any epidemiological cluster) was identified from the extended screening of 4,200 bloody diarrhoea specimens. Clearance for most cases was between 7 and 84 days (mean: 43 days) however STEC O55:H7 continued to be detected in the faeces of an asymptomatic 7-year old case for 10 months.

#### Whole genome sequencing

**Figure 2:**
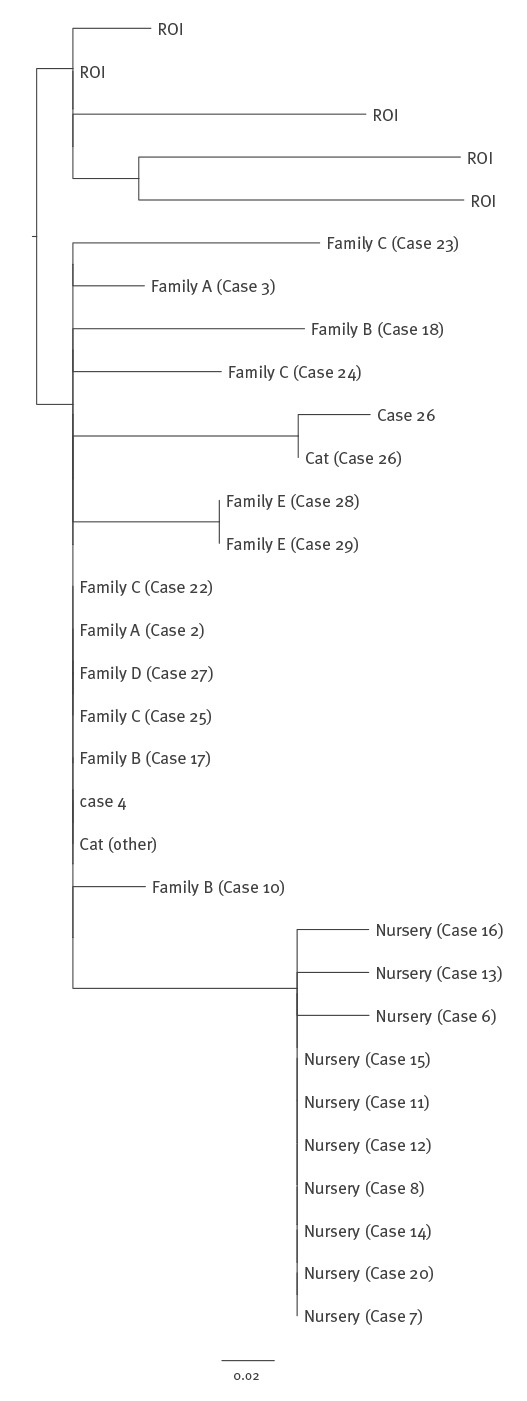
Maximum likelihood phylogeny of STEC O55:H7 isolates showing the monophyletic cluster associated with the outbreak cases and closely related strains from the Republic of Ireland

ROI: Republic of Ireland; SNP: single nucleotide polymorphisms; STEC: Shiga toxin-producing *Escherichia coli*.

All Dorset isolates from 24 cases and two cats were similar to each other forming a distinct clade within five SNP differences of the outbreak strain of STEC O55:H7. They were very similar (1–12 SNPs) to six Irish isolates (five were within 5 SNPs) from 2013 to 2014 but relatively distant (> 250 SNPs) from the five earlier Irish isolates. Isolates linked to the nursery were more closely related to each other than to isolates from the earlier cases in 2014 (Figure 2). Despite a period of almost 4 weeks between symptomatic cases in the nursery, phylogenetic analysis showed the isolates from three cases from weeks 47 and 48 in 2014 were identical to the isolate from the case identified in week 43, consistent with human-to-human transmission. Case 1 and the primary nursery case (Case 5) were diagnosed by serology therefore no isolate was available for sequencing.

The phylogeny was constructed based on 38 core genome SNPs with all isolates within 5 SNPs of each other.

### International investigations

#### Other STEC O55 isolates

All Irish cases were living in counties along the east coast and there were no common exposures between previous Irish cases occurring during 2012–14. There were no epidemiological links between the Dorset cases and the Irish cases. With the exception of the isolates from ROI, other STEC O55 isolates reported elsewhere in Europe in 2014 had different microbiological characteristics than the Dorset cluster (different serotype, *stx* profile and ability to ferment sorbitol) thus were not included in this investigation. There were three *E. coli* O55 cases elsewhere in England reported during the period of the outbreak who did not meet the outbreak case definition. One was STEC O55:H9 *stx*2d, another was a returning traveller who had positive serology only for *E. coli* O55 and therefore the strain could not be identified whilst the third case was STEC O55:H7 but was 10 SNPs apart from the outbreak WGS cluster and had no epidemiological link to Dorset.

#### Animal movement

Bird populations remained constant in the south west and south east England from 2013 to 2015, suggesting no new risks. Teal and black-headed gulls were the only species identified where there was significant level of movement between ROI, south England and Europe. Black headed gulls are common in Great Britain and there are large colonies along the south and east coasts of England.

**Figure 3:**
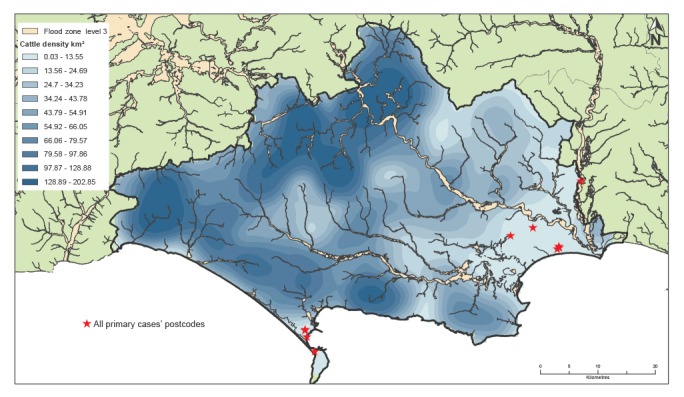
Map of Dorset cattle density, major rivers, flood zones and (co) primary cases’ postcodes, outbreak of Shiga toxin-producing *Escherichia coli* 055:H7, Dorset, July 2014−September 2015

A total of 1,149 cattle from ROI were moved to 69 separate premises in Dorset in the two year period between 2013 and 2014. These premises are located mainly in the north and west of the county, away from, but upstream of, the residences of (co)primary cases in the south of the county near the coast (Figure 3).

Crown Copyright and database rights 2015. Ordnance Survey Licence number 100051110.

^a^ Density as at January 2014.

Cattle data taken from Radar 10/12/15. Cattle Tracing System number of cattle on first day of month. Kernel Density (search radius 6 km, cell size 500 m).

### Control measures and communication

We managed cases and contacts using national STEC guidance [18], including appropriate exclusion from work, school or childcare of confirmed cases and contacts at increased risk of transmission, until shown microbiologically to have cleared the infection [18]. We provided advice on hand and food hygiene together with guidance on environmental cleaning and disinfection to cases and contacts and to venues such as a petting farm, nursery and schools. Staff and children were screened at the nursery; a measure that is not in the national guidance but has been used in other outbreaks [23]. In total, eight children were excluded for a period of time ranging from 7 to 84 days until microbiological clearance.

The nursery with the cluster of cases closed voluntarily on 26 November 2014 to facilitate screening of staff and children and deep cleaning of the premises.

Proactive public messages initially focused on basic infection control measures but became more specific, advising people to be extra vigilant with hand hygiene before preparing food and after contact with pets and animals. In response to widespread media interest, including coverage criticising PHE’s handling of the outbreak, the regional BBC Health Correspondent was offered a full access briefing including a visit to PHE laboratories. This resulted in factually accurate reporting and reiteration of the public health messages in a BBC television documentary in November 2015, with content promoted on its Facebook page [24].

## Discussion

In addition to being the first recorded outbreak of STEC O55:H7 in England, striking features of the outbreak include its seasonality, the high proportion of cases developing HUS (13/31) compared with the background rate for STEC O157 (5%) [2] and the geographical component linking all (co)primary cases to Bournemouth and Weymouth in Dorset. Evidence of human-to-human transmission of STEC O55:H7 within the nursery cluster was shown by the propagated epidemic curve and WGS with cases in this cluster forming a largely homogenous monophyletic sub-cluster among isolates in the outbreak as a whole. Overall, isolates showed more diversity than would be expected in a STEC point-source outbreak [25]. The genetic similarity between isolates taken from Dorset cases and the 2013–14 Irish cases suggests infection by the same or closely related populations of STEC O55:H7 and may represent a common zoonotic reservoir. Enhanced surveillance suggested infection to be less widespread than feared in the Dorset population. Contacts were tested more widely than normal (for a STEC outbreak) using culture/PCR and/or serology ensuring the identification of asymptomatic household contacts. To date there have been no further cases in this WGS cluster, although other *E. coli* O55 cases have occurred elsewhere in England. Despite the use of an iterative re-interviewing process, no common exposures linked all primary cases and food, water and environmental testing was negative for contamination by STEC O55:H7. There was little evidence to support the hypotheses of contaminated food items or drinking water or a specific recreational/environmental exposure. Given this, and the geographical clustering around two areas of Dorset, the third hypothesis of a local endemic zoonotic infection acquired in humans from infected pets and/or directly from the environment seems the most likely cause of this outbreak.

Due to the protracted nature of the outbreak, problems in recall may have hampered the effectiveness of the iterative re-interviewing approach, given the time lag between cases. It is possible that using the onset date of the associated primary case as the starting point for a 14-day exposure history-taking among asymptomatic cases missed important exposures if their infection had been acquired earlier. However, all of the asymptomatic cases were picked up through screening of contacts or nursery screening, all those identified by serology were epidemiologically linked to a WGS-confirmed case and many asymptomatic cases were adult household contacts of children, so it is likely that the majority of them were secondary cases whose infection was acquired by human-to-human transmission. Even if putative exposures had been identified, having only ten (co)primary cases would have made hypothesis testing via an analytical study challenging.

The environmental hypothesis appears most likely although there were no positive environmental samples (including boot socks, animal faeces, bird sanctuaries or water) to confirm it. The delay between symptom onset and environmental sampling could have reduced the chance of a positive finding and sensitivity of direct culturing from faeces can be poor if STEC numbers are low. Similar delays may also have reduced the likelihood of finding a positive sample among pets. There is currently no evidence of STEC O55:H7 in the cattle population in the United Kingdom but cattle and other ruminants are a known source of STEC [26]. Delays between infection and sampling, particularly among screened contacts, may also have led to cases being missed early in the outbreak. However, after the first few cases we tested contacts more widely than normal for a STEC outbreak and detected a number of asymptomatic household contacts using serology, so we are confident that any symptomatic contacts would also have been identified microbiologically, either via culture or serology. Furthermore, it is possible that asymptomatic or mild diarrhoeal illness may have been underestimated as most case ascertainment of STEC O55:H7 was due to presentation with HUS, with non-HUS-associated infections being missed apart from the periods of enhanced surveillance. The absence of additional cases arising from the testing of all diarrhoeal specimens from local and regional laboratories for STEC O55:H7 over a 12-week period is evidence against this, however, this extended testing regime occurred in the autumn, when fewer or no cases were expected.

STEC O55:H7 is phylogenetically closely related to STEC O157:H7 [12,25] and assumed therefore to have similar exposure risks and transmission routes. The observed seasonality (July–October 2014; May–September 2015) is similar to that seen for STEC O157 (April–September) in England and Wales [27]. This may reflect greater time spent outdoors during warmer months of the year, increasing exposure to the environment. Migratory birds as a transmission source common to Dorset and ROI could also help explain both the seasonality and genetic similarity between Irish and Dorset cases and birds (rooks) have previously been linked to STEC O157 infection in children [28]. The analysis focused on bird migration between ROI and Dorset but it is also possible that STEC O55:H7 was introduced by migratory bird species, with a shared winter feeding ground, that use the coast of ROI and the coast of England as destinations for summer feeding. In five of the six primary cases linked to Bournemouth, symptom onset occurred in a period of 1–9 days following flooding events, suggesting these events could have played a role. Three of these four cases reported visiting the public gardens or beach in Bournemouth and their illness could plausibly be explained by exposure to land recently flooded by stream water contaminated by animal faeces or to the sea into which the stream discharges. The STEC O55:H7 positive faecal specimens from two cats provided evidence that domestic pets may act as a vector for the pathogen and may support a hypothesis of an environmental or local zoonotic reservoir. This is consistent with reports of STEC O145 serotypes in sporadic HUS in children which were also isolated from their asymptomatic pet cats [29,30]. There were no common factors found for foods consumed by the pets, but it was not possible from the information collected to determine the extent to which cases’ pets interacted with the rural environment, wildlife or rodents. It is possible that there are several vectors responsible for the environmental spread and cases could also have acquired STEC O55:H7 directly from the environment.

This outbreak provides a number of learning points. Proactive media engagement was important and helped to ensure risk communication throughout the outbreak was effective and that the timing and control of information could be maintained by PHE. The evidence from WGS of isolates forming a closely related cluster was an important driver behind the extensive investigations undertaken. As WGS is used increasingly in England for outbreak investigation and detection, it is likely to be a driver in the future for investigating small outbreaks of illness that are not clearly linked epidemiologically [31,32].

The introduction of an additional SMAC agar plate into local laboratory processes helped reduce the number of cultures being sent to GBRU and enabled local detection of likely STEC O55:H7 in the absence of PCR. Enhanced surveillance through local laboratories, WGS, boot sock sampling and the combined use of hydrological and cattle census data are all methods that could be adapted and applied when investigating outbreaks of emerging pathogens with unclear aetiology. We conclude that although the cause of this outbreak remains elusive the varied investigations helped narrow the focus to a dispersed environmental source and/or a zoonotic reservoir.
